# Changes in microRNA (miRNA) expression during pancreatic cancer development and progression in a genetically engineered Kras^G12D^;Pdx1-Cre mouse (KC) model

**DOI:** 10.18632/oncotarget.5641

**Published:** 2015-10-19

**Authors:** Satyanarayana Rachagani, Muzafar A. Macha, Melanie S. Menning, Parama Dey, Priya Pai, Lynette M. Smith, Yin-Yuan Mo, Surinder K. Batra

**Affiliations:** ^1^ Department of Biochemistry and Molecular Biology, Fred & Pamela Buffett Cancer Center, University of Nebraska Medical Center, Omaha, NE; ^2^ Department of Biostatistics, Fred & Pamela Buffett Cancer Center, University of Nebraska Medical Center, Omaha, NE; ^3^ Department of Pathology and Microbiology, Fred & Pamela Buffett Cancer Center, University of Nebraska Medical Center, Omaha, NE; ^4^ Eppley Institute for Research in Cancer and Allied Diseases, Fred & Pamela Buffett Cancer Center, University of Nebraska Medical Center, Omaha, NE; ^5^ Department of Pharmacology and Toxicology, University of Mississippi Medical center, Jackson, MS

**Keywords:** miRNA, pancreatic cancer, KC mouse model

## Abstract

Differential expression of microRNAs (miRNAs) has been demonstrated in various cancers, including pancreatic cancer (PC). Due to the lack of tissue samples from early-stages of PC, the stage-specific alteration of miRNAs during PC initiation and progression is largely unknown. In this study, we investigated the global miRNA expression profile and their processing machinery during PC progression using the Kras^G12D^;Pdx1-Cre (KC) mouse model. At 25 weeks, the miRNA microarray analysis revealed significant downregulation of miR-150, miR-494, miR-138, miR-148a, miR-216a, and miR-217 and upregulation of miR-146b, miR-205, miR-31, miR-192, and miR-21 in KC mice compared to controls. Further, expression of miRNA biosynthetic machinery including Dicer, Exportin-5, TRKRA, and TARBP2 were downregulated, while DGCR8 and Ago2 were upregulated in KC mice. In addition, from 10 to 50 weeks of age, stage-specific expression profiling of miRNA in KC mice revealed downregulation of miR-216, miR-217, miR-100, miR-345, miR-141, miR-483-3p, miR-26b, miR-150, miR-195, Let-7b and Let-96 and upregulation of miR-21, miR-205, miR-146b, miR-34c, miR-1273, miR-223 and miR-195 compared to control mice. Interestingly, the differential expression of miRNA in mice also corroborated with the miRNA expression in human PC cell lines and tissue samples; ectopic expression of Let-7b in CD18/HPAF and Capan1 cells resulted in downregulation of *KRAS* and *MSST1* expression. Overall, the present study aids an understanding of miRNA expression patterns during PC pathogenesis and helps to facilitate the identification of promising and novel early diagnostic/prognostic markers and therapeutic targets.

## INTRODUCTION

Cancer is a compendium of perturbed genome functions and is characterized by the deregulation of several genes and their regulatory molecules, including microRNAs (miRNAs) [1, 2]. In general, miRNAs are 19–24 nucleotides long, noncoding RNA molecules that regulate the expression of 30% of protein-coding genes at the posttranscriptional level [3, 4]. These miRNAs are transcribed by RNA polymerase II as pre-miRNAs, which are processed by Drosha, to form hairpin-like intermediates called pre-miRNAs, which are approximately 70–100 nucleotides long and have two nucleotide overhangs at their 3′ ends [5–8]. Exportin-5 transports pre-miRNAs out of the nucleus and into the cytoplasm for further processing by dicer [9, 10], which converts the pre-miRNA into a 19–24 nucleotide long, double-stranded, mature miRNA [11]. The guide strand of mature miRNA gets incorporated into a complex to form the RNA-induced silencing complex (RISC), which recognizes the specific target mRNA through complementary base pairing, and consequently, either degrades or inhibits translation of the target mRNA [6, 12]. Recent reports have shown that miRNAs can play an important role in diverse biological functions such as development, normal cell physiology and pathological conditions like abnormal cell proliferation [13–15] and cancer [16, 17] including pancreatic cancer (PC).

Pancreatic cancer (PC) is the 10^th^ leading cause of cancer-related deaths worldwide and the fourth-leading cause of cancer-related deaths in the United States [18–21]. PC has a five-year survival rate of less than 6% [20]. This high mortality rate among PC patients is due to the lack of early symptoms, diagnostic and prognostic markers, metastatic disease at the time of clinical diagnosis, poor response to therapy, and a high rate of recurrence [22]. Thus, in order to identify suitable early diagnostic markers or therapeutic targets to combat this disease, there is an urgent need to understand the pathogenesis of PC. Several studies have shown the abnormal expression of miRNAs including miR-21, Let-7b, miR-100, miR-217, and miR-216 in PC and have proposed them as candidates for early diagnosis and potential molecular targets [23, 24]. However, due to unavailability of early stage PC biopsies from humans, the stage-specific deregulation of miRNA during PC progression is largely unknown.

In this study, we investigated the expression profile of miRNAs at various stages of PC progression using the Kras^G12D^;Pdx1-Cre mouse model. This model is histopathologically recapitulates human PC [25] and enables one to monitor the progression of PC from the onset of precursor lesions to cancer development. Hence, understanding the role of miRNA in PC pathogenesis using this model will subsequently help to develop early detection markers, as well as therapeutic and preventive strategies for PC. Further, this study examined the expression of various components of the miRNA biosynthetic machinery and some of their selected target genes for differentially expressed miRNAs. Findings were extrapolated to human PC tissues and cell lines by investigating the expression of differentially expressed miRNAs as observed in the Kras^G12D^ mouse model. The overall objective of this study was to establish a global differential expression profile of miRNAs during the course of initiation and progression of PC. Moving forward, the identified miRNAs can be used to create novel biomarker(s) for validation studies and therapeutic targets to combat this lethal disease.

## RESULTS

### Pancreatic cancer progression model

Our previous study using the Kras^G12D^;Pdx1-Cre (KC/floxed Kras^G12D^) PC mouse model showed the presence of pancreatic intraepithelial neoplasia (PanIN) lesions as early as 10 weeks of age; these lesions progressed to pancreatic ductal adenocarcinoma (PDAC) and metastasized to the liver, lungs, and intestines by 50 weeks of age [26]. We observed PanIN-I lesions at 10 weeks of age that progressed to PanIN I, II, III lesions at 25 weeks of age, replacing larger portions of the pancreatic parenchyma [26]. At 40 weeks of age, the majority of the parenchyma was replaced by PanIN III and extensive desmoplasia, whereas at 50 weeks of age, animals replaced the pancreatic parenchyma with full-blown PDAC, and metastatic spread involving the colon, liver, and lungs in 60–70% of the animals [26].

### miRNA microarray

In order to identify the deregulated miRNAs during PC progression, we used affymetrix miRNA microarray to analyze the global miRNA expression in the pancreas of 25-week old KC animals (Kras^G12D^;Pdx1-Cre) and its age-matched littermate control mice (LSL Kras^G12D^). We chose 25-week old mice as the base line because, at this time point, the mice had only developed PanIN I, II, and III lesions but not PDAC. Hence, this age represented the stage prior to the onset of PC.

**Figure 1:**
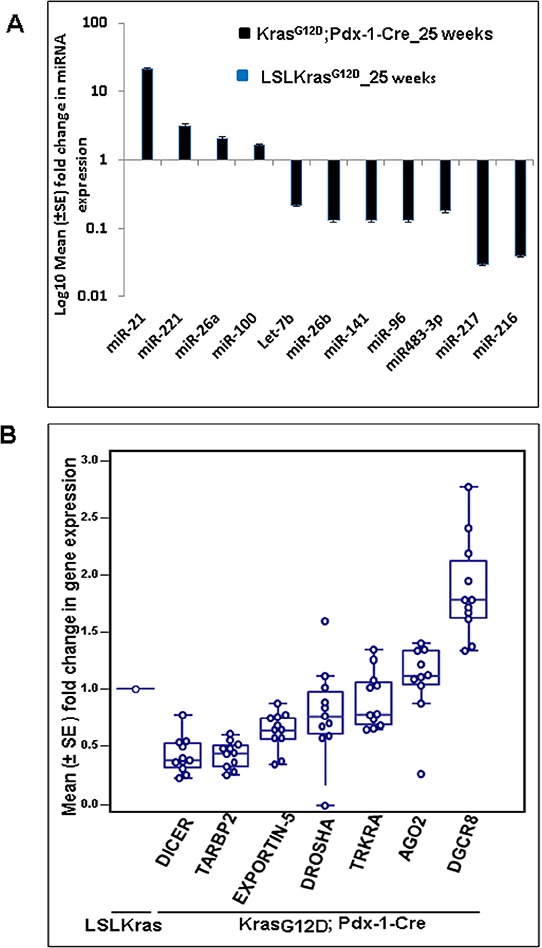
The miRNA array revealed several upregulated and downregulated miRNAs in Kras^G12D^; Pdx-1-Cre mice at 25 weeks of age **A.** Validation of a few upregulated and downregulated miRNAs was confirmed by real-time PCR analysis, using TaqMan assays specific for individual miRNAs. U6 snRNA was used as an internal control for normalization. **B.** Real-time PCR analysis of miRNA biosynthetic pathways in KrasG12D;Pdx1-Cre and LSL- Kras^G12D^ animals at 25 weeks of age using specific primers. Mouse β-actin was used as an internal control for normalization. The fold change was calculated using the ΔΔ^Ct^ method.

Differentially expressed miRNAs were identified using QC tool software. The miRNA microarray analysis for the KC animals compared to controls revealed that miR-150, miR-494, miR-138, miR-148a*, miR-216a, and miR-217 (*p-value* = 0.01) were significantly downregulated (Table 1), whereas, miR-146b, miR-205, miR-31, miR-192, and miR-21 (*p-value* = 0.01) were significantly upregulated (Table 2). A majority of the miRNAs were downregulated compared to the number of miRNAs that were upregulated in the KC animals (Supplementary table S1). The panel of differentially expressed miRNAs were validated by real-time PCR using TaqMan assays, and the results were consistent with the miRNA microarray data that showed up-regulation of miR-21, miR-221, miR-100 and miR-26a and down-regulation of miR-26b, miR-141, miR-96, miR483-3p, miR-216, and miR-217 in the KC compared to control mice (Figure 1A).

**Table 1:** Top/Significantly downregulated miRNAs in Kras^G12D;^Pdx1-Cre mice

S.No	microRNA	Fold change	predicted Target genes
1	miR-150	0.149	c-Myb, Eif4b&e, Ephb2, Elk1, Mcc
2	miR-494	0.293	Fgfr2, Cdk6, Nfat5, Ccnt2, IGF1R, Fgf7, Ccnd2, Socs6, Bmpr2,
3	miR-138	0.301	Sin3a, Rmnd5a, Thrb, Dek, Rhoc, Nfib, Hif1a, Tjp1
4	miR-148a*	0.351	Bcl2l11, fbn1, Itga5,9&11, Dgcr8, Snn, Dicer1, E2f3&7, Tgfbr1, wnt1, Itga1
5	miR-193	0.351	Kit, Tgfb2, Ets1, Etv6, Tgfbr3, Kras, Fgf1, Tcf4.
6	miR-451	0.365	Psmb8, Mex3c, Cab39, Fbxo33, Gpr77
7	miR-216a & b	0.406 & 0.447	Jnk2, Esr1, Sp4, Mmp16, Smad7, Tssc1, Tgfbr2, Sox6, Grb2,
8	miR-29b	0.409	Eln, Col3a1, Col4a5, Col5a1, Has3, Igf1, Dnmt3a, Camk4, Hdac4, Vcl, Ccnd2, Nkrf, Dicer1, Sp1, Ncoa3
9	miR-375	0.550	Max protein (Max)
10	miR-217	0.548	Kras, Esr1, Etv6, Dek,

**Table 2:** Top/Significantly up regulated miRNAs in Kras^G12D;^Pdx1-Cre mice

S.No	microRNA	Fold change	Some of predicted Target genes
1	miR-146b	18.46	MMP16,
2	miR-205	9.89	Cdh11, Cdkn1b, E2F1
3	miR-31	9.50	St7, Pdcd11, E2f2, Ret, Dicer1, Pcdha4-12,
4	miR-192	8.35	Cdc7, Ercc3, Pim1, Mcm10, Hoxa10, Mad2L1, PRPF38A, Racgap1, and Smarcb1
5	miR-194	7.11	IGF1R, Stat1, ITGA1, Sox11, Lats1
6	miR-21	5.76	Tgfbr2, Tgfbi, Sox2, Sox5, Sox7, PTEN, TPM1, PDCD4, Maspin, Rasa1&2, Cstf3
7	miR-379	5.37	Insr, Igf1r, Gdf6, Eif4g2, Edn1, Nfat5
8	miR-214	4.13	Arhgap28, Gnao1, Nr2c1
9	miR-541	4.57	Gab1, Dcc, Braf1, Tgfbr3
10	miR-199b	3.41	Itga1,3&8, fgf7,10&16, Rb1, Ppp2r2a, Ela1, Ppp2r5e, Fn1, Sp1, Met, Igf1, Zab1,

The downregulation of the majority of miRNAs led us to investigate the expression status of the miRNA biosynthetic machinery during the progression of PC using the KC mouse model. Real-time PCR analysis revealed that expression of Dicer, Drosha, Exportin-5, TRKRA, and TARBP2 were downregulated, while expression of DGCR8 and Ago2 were upregulated in KC mice compared to control littermates (Figure 1B).

**Figure 2:**
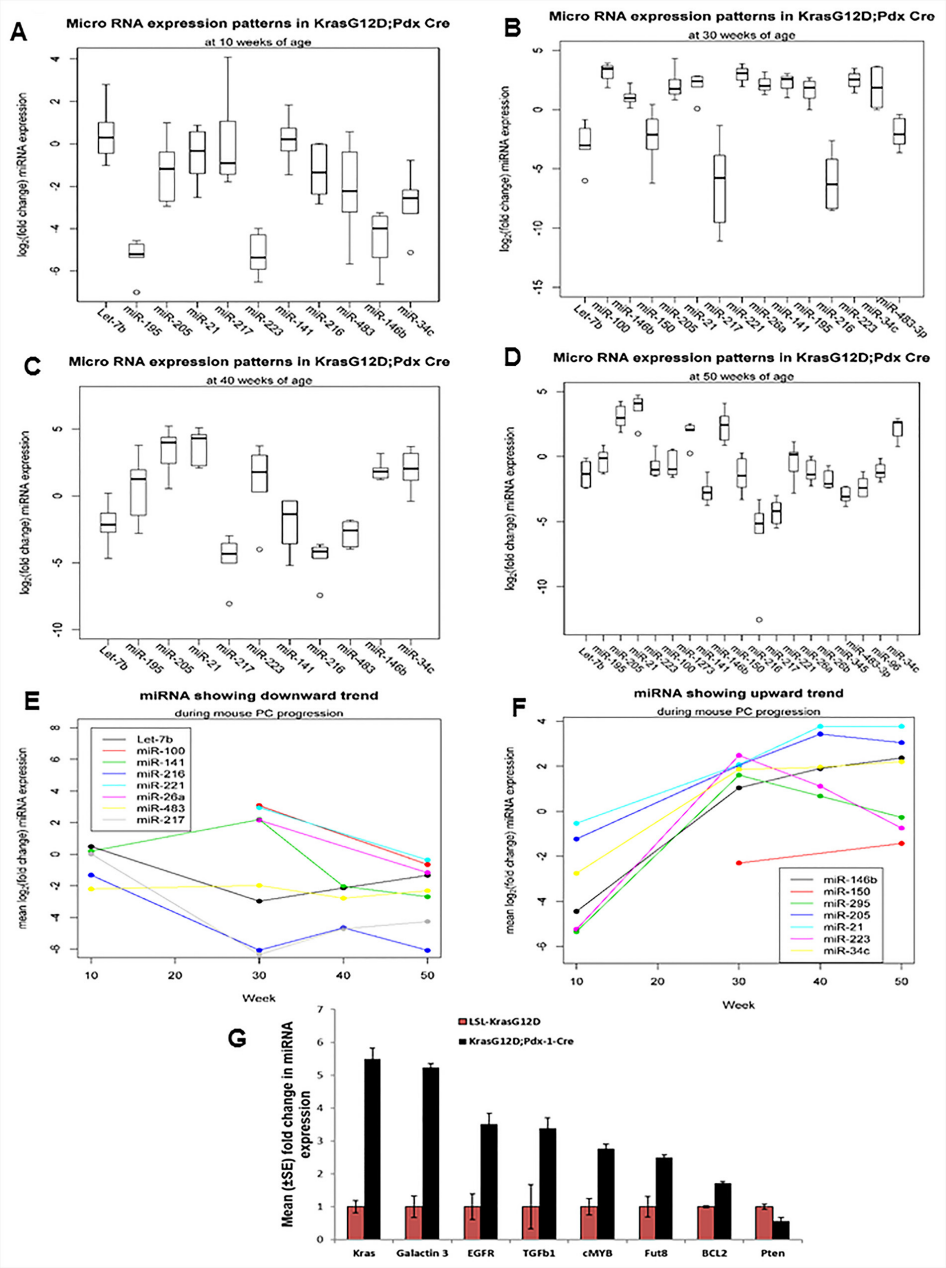
Expression profiles of miRNAs during the mouse PC progression in KrasG12D; Pdx1-Cre mice and their contemporary littermate animals **A, B, C, D.** Expression patterns of miRNA at 10, 30, 40, and 50 weeks of PC progression was analyzed by real-time PCR, using TaqMan assays specific for individual miRNAs. U6 snRNA was used as an internal control for normalization. The fold change was calculated by ΔΔ^Ct^ method. **E.** and **F.** Showing trends of miRNA expression during the mouse PC progression.

The top differentially deregulated miRNAs were further analyzed at 10, 30, 40, and 50 weeks of PC progression. At 10 weeks of age, expression of miR-141 and Let-7b were upregulated, but their expression was not statistically significant. On the other hand, miR-146b, miR-34c, miR-223, miR-195 (*p-value* = 0.031) and miR-216 (*p-value* = 0.063) were downregulated in KC mice compared to control littermates. However, no significant difference was observed in the expression of pancreas-specific miR-217 (Figure 2A). At 30 weeks of age, the expression of miR-216 (*p-value* = 0.016), miR-217 (*p-value* = 0.0078), miR-150 (*p-value* =0.023), Let-7b (*p-value* = 0.031,) and miR-96 were significantly downregulated, whereas the expression of miR-146b (*p-value* = 0.0078), miR-205, (*p-value* - 0.0078), miR-21, miR-195 (*p-value* = 0.031), and miR-34c (*p-value* = 0.063) were significantly upregulated in KC animals compared to control animals (Figure 2B). At 40 weeks of age, the expression of miR-216, miR-217, miR-223, miR-141, miR-483-3p (*p-value* = 0.031), miR-195, Let-7b (*p-value* = 0.063) and miR-96 were significantly downregulated; on the other hand, the expression of miR-21, miR-205, miR-146b (*p-value* = 0.031), and miR-34c (*p-value* = 0.063) were upregulated in KC mice compared to the control animals (Figure 2C). Further, at 50 weeks of age, the expression of miR-216, miR-217, miR-345, miR-141, miR-483-3p, miR-26b, miR-96, Let-7b (*p-value* = 0.01), miR-100, miR-26a and miR-150 (*p-value* = 0.094) were further downregulated in KC animals compared to control mice (Figure 2D). The expressions of miR-216 and miR-217 were also progressively reduced in KC mice, but the expressions of miR-21, miR-205, miR-146b, miR-34c, and miR-223 progressively increased (Figure 1A, 2A–2D). At 50 weeks of age, variation in expression of miR-221 was not statistically significant between the KC and control animals (Figure 2D). The overall trends of miRNA expression during the mouse PC progression model are shown in Figures 2E and 2F.

### Validation of miRNA target genes

In order to confirm the expression of genes targeted by the differentially expressed miRNAs in PC, first we performed *in silico* analysis using an online bioinformatics program, mirecords/miRDB, which utilizes miRanda, Target scan, Pic Tar, *etc*., to predict several potential targets genes (Table 1 and Table 2). A few of these target genes, that are important in PC development, were analyzed by Real time PCR at 50 weeks of age. The expression of Kras (5-fold), Galectin-3 (5-fold), EGFR (3.5-fold), TGFβ1 (3.5-fold), cMyc (2.8-fold), Fut8 (2.5-fold), and Bcl2 (2-fold) were significantly upregulated, while the expression of PTEN was significantly downregulated in KC animals compared to controls (Figure 2G).

### Expression patterns of miRNAs in human PC cell lines

**Figure 3:**
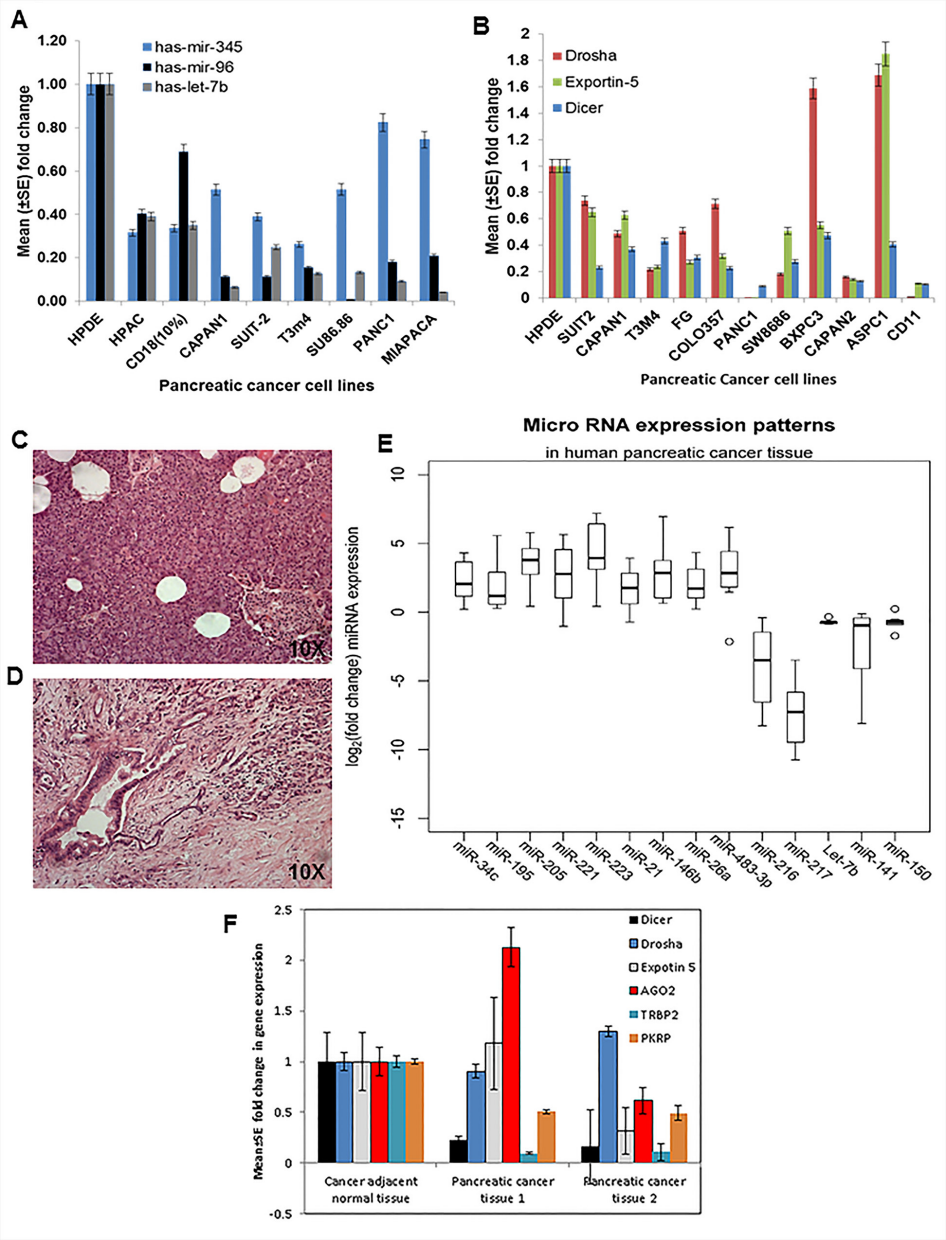
Expression patterns of miRNAs and components of the biosynthetic machinery in human PC tissues and cell lines **A.** Expression profiles of miRNA in human PC cell lines using TaqMan assays specific for individual miRNA based on real-time PCR analysis. RNU6B was used as an internal control for normalization. The fold change was calculated by using the ΔΔ^Ct^ method. **B.** Components of the miRNA biosynthetic pathway in human PC cell lines by using specific primers for each gene by real-time PCR analysis. Human β-actin was used as an internal control for normalization. The fold change was calculated by using the ΔΔ^Ct^ method. **C.** H & E staining of cancer-adjacent normal tissue. **D.** PC tissue and **E.** expression patterns of miRNA in human PC tissue and adjacent normal tissue using real-time PCR using TaqMan assays specific for individual miRNA. RNU6B was used as an internal control for normalization. The fold change was calculated by using the ΔΔ^Ct^ method. **F.** Components of the miRNA biosynthetic pathway in human PC tissue and adjacent normal tissue were determined using specific primers to each gene by real-time PCR analysis. Human β-actin was used as an internal control for normalization. The fold change was calculated by ΔΔ^Ct^ method.

In order to extrapolate the mouse miRNA microarray data to human PC, we next checked the expressions of a few differentially expressed miRNAs in eight human PC cell lines, as well as in the immortalized normal pancreatic ductal cell line (HPDE). Results showed the downregulation of miR-345, miR-96, and Let-7b in the majority of the PC cell lines compared to the HPDE cells (Figure 3A), indicating that these miRNAs were expressed in ductal cells rather than in tumor stroma. Also, the expression analysis of miRNA biosynthetic machinery in PC cell lines showed significant downregulation of Drosha, Dicer, and Exportin-5 compared to the HPDE cell line (Figure 3B).

### Expression patterns of miRNAs in human PC tissue

The differentially expressed miRNAs that were identified from mouse miRNA array data were further analyzed in eight samples of human PC tissues (Figure 3C) and their corresponding eight adjacent normal tissue (Figure 3D). The expression of miR-223, miR-483-3p (*p-value* = 0.01), 146b, 205 (*p-value* = 0.001), 221, 21 (*p-value* = 0.023), 195, 34c and miR-26a (*p-value* = 0.0078) were significantly upregulated, whereas the expression of miR-216, miR-141, miR-217, Let-7b (*p-value* = 0.001), and Let-150 (*p-value* = 0.01) were significantly downregulated in human PC tissues as compared to the cancer-adjacent normal tissues (Figure 3E). Additionally, the expression of proteins involved in miRNA biosynthesis, such as Drosha, Dicer, and Exportin-5, were analyzed and found to be significantly downregulated in human PC tissues as compared to the cancer-adjacent normal tissues (Figure 3F).

### Ectopic overexpression of Let-7b in human PC cell lines

**Figure 4:**
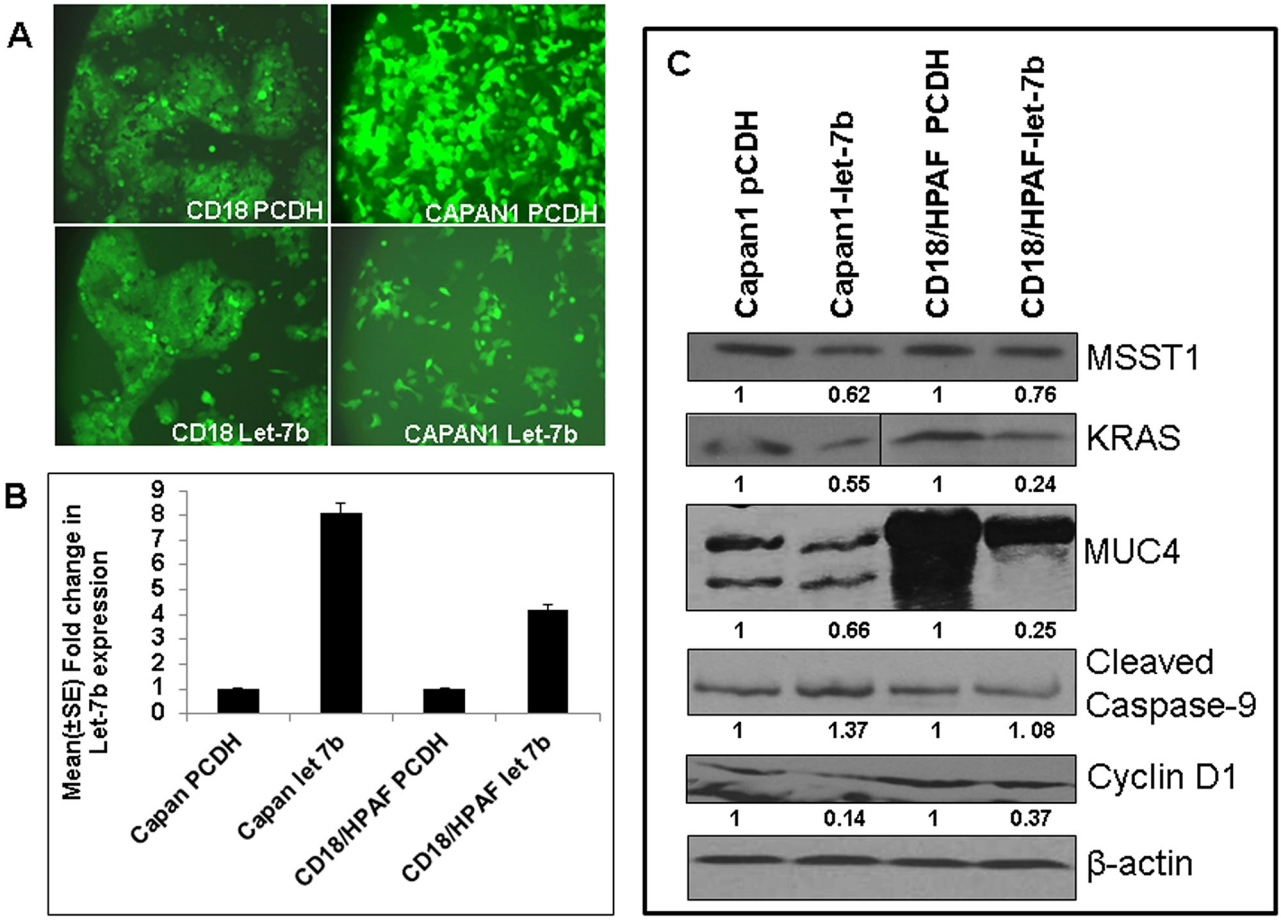
Overexpression of Let-7b in human PC cell lines **A.** PC cells infected with lentivirus carrying Let-7b miRNA/control, along with GFP as a selection marker, revealed 100% PC cells expressing GFP, indicating overexpression of Let-7b/control vector in PC cell lines. **B.** Validation of Let-7b overexpression in GFP-expressing PC cells was analyzed using the TaqMan assay by real-time PCR revealed significant upregulation of Let-7b in human PC cell lines compared to control vector transfected PC cells. RNU6B was used as an internal control for normalization. The fold change was calculated by using the ΔΔCt method. **C.** Immunoblotting of protein lysates collected from PC cell lines ectopically overexpressing Let-7b and its control cells revealed downregulation of Kras, MSST1, MUC4 and Cyclin D1, and upregulation of cleaved caspase-9 (target genes predicted by *in silico* analysis). β-actin was used as a loading control.

The role of Let-7 family members and their target genes are well known in various cancer [27]. Previous studies have shown that restoration of Let-7 results in downregulation of oncogenic Kras, leading to inhibition of cell proliferation and activation of the mitogen-activated protein kinase [28]. Similarly, HMGA2 is a direct target of Let-7 family members [29–31]. Our results in the KC mouse model also showed downregulation of Let-7b during PC progression. Further, our *in silico* analysis of Let-7b revealed several target genes like *MUC4, NCOA3, KRAS, HMGA2,* which are critical in PC pathogenesis. Herein, we overexpressed Let-7b and its scramble vectors in two PC cell lines, CD18/HPAF and Capan1, by infection with a lenti-viral supernatant (collected after 48 and 72 hours post transfection of 293FT cells) after mixing with 4 μg/ml of polybrene and analyzing its effect on downstream targets. The green fluorescent protein (GFP)-positive cells were sorted by Fluorescence Activated Cell Sorting (FACS) (Figure 4A); Let-7b overexpression was confirmed by TaqMan assays using real-time PCR (Figure 4B). We observed eight-fold and four-fold increase in the expression of Let-7b in Capan 1 and CD18/HPAF cells, respectively, compared to vector control cells (Figure 4B). Our results from western blot analysis revealed downregulation of MUC4, KRAS, MSST1, and Cyclin D1, and upregulation of caspase-9 in Let-7b overexpressing CD18/HPAF and Capan1 PC cells compared to vector transfected control cells (Figure 4C); β-actin was used as a loading control. These findings suggest that downregulation of Let-7b plays an important role in PC progression.

## DISCUSSION

Due to unavailability of early stage samples from human PC patients, we utilized the well-characterized spontaneous mouse model (KC) of PC [32–36] to analyze the global miRNA expression profile. Notably, this model histopathologically recapitulates human PC. Using this PC model, we and others have observed PanIN lesions at as early as 10 weeks of age, as well as PDAC with metastasis (in 60–70% of KC animals) to distant organs by 50 weeks of age [26, 35]. Although high-grade PanINs (PanIN III) were rarely observed before 20 weeks of age, their frequency increased progressively with age (25–50 weeks); however, low- and high-grade PanINs were observed starting at 25 weeks of age. Therefore, the present study analyzed the global miRNA expression profile at 25 weeks of age, when the majority of acinar cells were replaced by high-grade PanINs and marked desmoplasia [26].

Using miRNA microarray analysis, we identified several differentially expressed miRNAs in KC animals compared to control mice, with most of the miRNAs having tumor suppressive functions (Tables 1 and 2). The microRNA microarray revealed significant downregulation of miR-494 at 25 weeks of PC progression in the KC mouse model. A recent study has also shown significant downregulation of miR-494 in human PC tissues compared to non-tumor tissues, correlating with PC metastasis and decrease free survival in patients. Furthermore, decreased expression of miR-494 is due to loss of SMAD4 in PC. Ectopic expression of miR-494 in PDAC cells resulted in downregulation of FOXM1, inhibition of β-catenin nuclear translocation, decreased cell proliferation, migration, invasion, and greater sensitivity to gemcitabine [37]. The expression of pancreas-specific tumor suppressors miR-217 and miR-216 were unaltered at 10 weeks of age (presence of PanIN-Ia and Ib), but progressively decreased from 25 – 50 weeks of age as PanIN lesions progressed to PDAC. (Figure 1A, 2A–2D).

In addition to KC mice, we also observed a significant downregulation of miR-216 and miR-217 in human PC tissue (Figure 3E); these results are in agreement with earlier studies on human PC [38–43] that show downregulation of miR-217 in 76.2% (16/21) of PC tissue as well as cell lines [43]. Similarly, the expression of miR-216 is significantly downregulated in PC [44]. Our *in silico* analysis revealed that miR-216 and miR-217 potentially target many important genes that play critical roles during the pathogenesis of PC (Table 1); and the downregulation of miR-217 [45] and miR-216 [44] suggests their potential as tumor suppressors in PC by targeting downstream targets, particularly the Kras oncogene [43] and Janus kinase 2 [44]. In addition to pancreas-specific miRNAs, we observed significant downregulation of miR-141 and Let-7b in human PDAC and in KC mice from 25–50 weeks of age compared to controls (Figure 1A, 2B–2D, 3E). Our human PDAC results were in agreement with earlier reports showing loss of Let-7b and miR-141 expression in PC samples compared to cancer-adjacent normal tissue [46, 47]. *In vitro* analysis of Let-7b overexpressed HPAF/CD18, and Capan1 PC cell lines showed significant downregulation of MUC4, Kras, MSST1, and Cyclin D1, and upregulation of cleaved caspase-9 (Figure 4C). Although the functional analysis of this Let-7b overexpression remains to be determined using our *in vitro* system, however previous studies have shown that overexpression of Let-7b in Capan1 cells resulted in decreased cell proliferation by downregulating Kras, Myc, and HMGA2, and activating mitogen-activated protein kinase [28–31, 46, 47]. In addition to affecting proliferation, the increased expression of cleaved caspase-9 in both HPAF/CD18 and Capan1 cells suggests it has a pro-apoptotic role that remains to be determined. Further, Kent *et al*. (2009) reported downregulation of miRNA-200 family members, including miRNA-141, in PC cell lines [48] and observed a positive correlation between the expression of miR-200 family members and E-cadherin expression, as well as a negative correlation with the zinc-finger E-box-binding homeobox 1 (ZEB1) [48, 49]. The transcription of miR-200 family members (miR-141 and miR-200c) is suppressed by ZEB1, which activates epithelial differentiation in PC, colorectal, and breast cancer cells. Epithelial-mesenchymal transformation (EMT) activators, such as transforming growth factor beta 2 and ZEB1, have 3′ UTR binding sites for miR-200 family members. Therefore, existence of a feedback loop between ZEB1 and miR-141 serves to stabilize the EMT process and regulate the invasion of cancer cells [49].

The present study also revealed significant downregulation of miR-150 during the progression of PC in both mice and humans (Figure 2A–2D, 3E). These results contradict a previous study on human PC [40], possibly attributable to species and/or PC stage variation. Similarly, the expression of miR-345 and miR-96 were significantly downregulated during the progression of PC in the mouse model (Figure 2D), which is consistent with previous reports [39, 40, 50]. However, a recent report in colorectal cancer patient tissues has shown significantly methylated mir-345 promoter region compared to the non-cancerous, adenoma, and normal colon tissues; further, ectopic over-expression of mir-345 in colorectal cancer cells significantly reduced cell proliferation by inhibiting the translation of anti-apoptotic BAG3 gene [51]. These studies suggest a pro-apoptotic function of miR-345; its downregulation during PC progression may be associated with promoter methylation.

The miR-26a miRNA inhibits the expression of c-myc, Cyclin D3 and E2, and cyclin-dependent kinases such as CDK4 and CDK6. Also, it stimulates CDK-inhibitors p14(ARF) and p21(CIP1) expression in an EZH2-dependent manner, suggesting an anti-proliferative role [52]. Accordingly, we observed significant downregulation of miR-26a in KC mice at 50 weeks of age, although it was upregulated at 25 weeks in KC mice and in human PC tissues compared to normal tissues (Figure 2B, 2D and 3E). Recently, significant downregulation of miR-26a was also reported in nasopharyngeal carcinoma tissues and cell lines (53); its ectopic expression has been shown to downregulate EZH2 expression, thereby inducing G_1_-phase cell-cycle arrest to decrease cell proliferation and colony formation (53). A decreased expression of miR-483-3p was observed during the progression of mouse PC, whereas it is overexpressed in human PC (Figure 2A–2D and 3E). These results are in agreement with previous reports that show miR-483-3p upregulation in PC tissue compared to the cancer adjacent normal tissue [53, 54]. Furthermore, they showed that ectopic expression of miR-483-3p significantly inhibited the expression of DPC4/Smad4 protein in PC cell lines, leading to increased cell proliferation and colony formation *in vitro* [53, 54]. The miRNA microarray also revealed decreased expression of miR-148a and miR-451 in mouse PC, an observation consistent with other reports [40, 41, 55–58]. Similarly, another study showed that the expression of miR-148a/b and miR-375 were significantly downregulated in the PC developed from p48Cre;Kras^G12D^ transgenic animals compared to the normal controls [59].

Several studies have demonstrated the upregulation and importance of miR-21 in PC patients. Although the expression of miR-21 expression does not correlate with tumor size, differentiation, nodal status, or T stage, its overexpression was associated with poor patient outcomes and overall survival compared to patients without miR-21 or patients with faint/focal expression in node-negative disease [60, 61]. Overexpression of miR-21 is associated with both high Ki67 proliferation index and liver metastasis [62], whereas inhibition of miR-21 expression in MiaPaCa-2 PC cells resulted in decreased cell proliferation, increased cell death, and increased expression of the tumor suppressor gene like programmed cell death 4 (PCD4) gene [63]. In addition, miR-21 has been shown to suppress the activity of the tumor suppressor gene tropomyosin 1 (TPM1), thereby promoting tumorigenesis [64]. In line with these reports, our study also indicates the progressive increase in miR-21 and miR-205 from 10 to 50 weeks of age in KC mice compared to the control animals (Figure 1A and 2A–2D). Similarly, we also observed upregulation of miR-21 and miR-205 in human PC samples compared to cancer- adjacent normal tissue (Figure 3E). Several reports have also shown elevated expression of miR-21 and miR-205 in a panel of PC cell lines and tissues compared to the normal controls [40–42, 50, 55, 60, 65–68]. Rieu *et al*. also reported in the KC animals, progressive increases in miR-21 and miR-205 expression from PanIN lesions to full-blown PDAC, with strongest expression of miR-21 in precursor lesions with phenotypic changes in the ducts [69]. Further, Rieu *et al*. have shown that expression of miR-21, miR-221, miR-222, and Let-7a increases with PanIN grade, with the strongest expression in the hyperplastic ducts with PanIN 2/3 lesions [69]. Similarly, the expression of miR-10, miR-21, miR-100 and miR-155 was shown to increase in p48-Cre/Kras^G12D^ mice when compared to control animals [59]. We observed downregulation of miR-146b, miR-34c, and miR-223 at 10 weeks of age; however, their expression increased with the progression of PC in KC animals compared to control animals (Figure 2A–2D). We also observed upregulation of these miRNAs in the human PC samples (Figure 3E). Notably, the expression of miR-34c is activated by p53 following DNA damage and serves as an important regulator of c-Myc expression, acting downstream to the p38 MAPK/MK2 pathway [70].

Deregulation of the miRNA biosynthetic machinery results in differential expression of miRNAs and downstream target genes. Abnormal expression of this machinery is associated with hematological malignancies [71], ovarian cancer [72], and neuroblastoma [73]. In conjunction with these reports, our results also revealed significant downregulation of many miRNA biosynthetic pathway components, such as dicer, TARBP2, and exportin-5, both during the progression of PC in KC animals (Figure 1B) and in PC cell lines and tissues compared to normal tissues (Figure 3B and 3F). The predicted target genes for differentially expressed miRNAs (Table 1 and Table 2) such as Kras (5-fold), Galectin-3 (5-fold), EGFR (3.5-fold), TGFβ1 (3.5-fold), cMyc (2.8-fold), Fut8 (2.5-fold), and Bcl2 (2-fold) were upregulated. On the other hand, PTEN was observed to be downregulated in KC mice, which highlights the importance of deregulated miRNA expression in the pathology of PC (Figure 2G). Both miR-216 and miR-217 act as potential tumor suppressors for PC by targeting the Kras oncogene [43].

In conclusion, the present study reveals the stage-specific (PanIN to PC) unique expression patterns for miRNAs, as well as miRNA biosynthetic machinery, during progression of PC. We have shown that in tumor samples compared to normal samples, the majority of miRNAs (miR-216, miR-217, miR-100, miR-345, miR-141, miR-483-3p, miR-26b, miR-150, Let-7b, Let-195 and miR-96) were downregulated, and few were upregulated (miR-146b, miR-205, miR-31, miR-192, miR-194 21, miR-379, miR-431, miR-541, and miR-199b). Future studies will focus on the analysis of stage-specific miRNA expression in the sera of mice collected during PC progression, as this is expected to provide the unique opportunity to develop early detection biomarkers for PC. Furthermore, restoring the expression of downregulated miRNAs in cancer cells may be performed to promote their de-differentiation and induce their reversion into a benign or normal cell. Overall, the current study offers the unique opportunity to develop early biomarkers for the diagnostic, prognostic and preventive management of PC.

## MATERIALS AND METHODS

### Experimental animals

The B6.129-*Kras^tm4Tyj^* (01XJ6) and B6. FVB-Tg (Ipf1-cre) 1Tuv (01XL5) mice were obtained from the National Cancer Institute (NCI) Mouse Models of Human Cancers Consortium (MMHCC, Frederick, MD, USA). In order to decipher miRNAs in early PC lesions, we obtained the PC progression model by crossing LSL-Kras^G12D^ animals [74] with Pdx-1-Cre [35]. The animals positive for both Kras and Pdx Cre (Floxed Kras^G12D^) and their contemporary littermates unfloxed Kras (LSL Kras^G12D^) from the F1 litter were sacrificed at 10, 25, 30, 40, and 50 weeks of age (eight animals for each group at each time point). The pancreas was resected, and half of the pancreas was flash frozen in liquid nitrogen (total RNA and miRNA isolation); the other half was fixed in 10% buffered formalin and embedded in paraffin. Serial tissue sections were cut (4 μM) and stained with hematoxalin and eosin. Throughout the study, the animals were subjected to a 12 hours of dark/light cycle with food and water *ad libitum*. Animal studies were performed in accordance with the United States Public Health Service “Guidelines for the Care and Use of Laboratory Animals” under an approved protocol by the University of Nebraska Medical Center's Institutional Animal Care and Use Committee (IACUC).

### Isolation of total RNA and miRNAs

Total RNA and miRNA were isolated from the pancreas of floxed Kras^G12D^ mice positive for Kras;Pdx-1-Cre and unfloxed LSLKras^G12D^ mice, human PC cell lines, tissues and their cancer-adjacent normal tissues (eight tissues each) using mirVana™ miRNA Isolation Kit (Applied Biosystems/Ambion, Austin, TX, USA). The RNA concentration was measured by Nanodrop Spectrophotometer (NanoDrop Technologies Inc., Wilmington, DE, USA), and the quality was analyzed using bioanalyzer (Agilent technologies, Waldbronn, Germany). Samples with good integrity were used for miRNA array, real-time PCR, and TaqMan assays.

### Affymetrix microRNA microarray

GeneChip^®^ miRNA Array (Cat. No: 901325) was used to study the global expression profile of miRNAs at 25-week of Kras^G12D^;Pdx-1-Cre mice and LSLKras^G12D^ mice. Two micrograms of total RNA from two floxed Kras^G12D^ (Kras^G12D^;Pdx1-Cre) and two unfloxed LSL-Kras^G12D^ (LSL Kras^G12D^) animals were labeled with the FlashTag Biotin RNA Labeling Kit for Affymetrix GeneChip^®^ miRNA arrays (Genisphere, Hatfield, PA, USA) and hybridized on Affymetrix GeneChip^®^ miRNA arrays (Affymetrix, Santa Clara, CA, USA). Hybridization, washing, and scanning of the slides were done according to Affymetrix's recommendations (Fluidics Protocol FS450_0003). Data was extracted from the images, quintile normalized, summarized (median polish) and log2-transformed with the miRNA QC tool software from Affymetrix.

### Detection of mature miRNAs by TaqMan assays

The TaqMan miRNA assay kits purchased from Applied Biosystems were used for expression and validation analysis of miRNAs during the progression of PC in both mice as well as human tumor samples. In order to analyze the expression patterns, first, reverse transcription reactions for each miRNA was performed using 1.5 μl of 10X reverse transcription buffer, 1.0 μl of MultiScribe™reverse transcriptase (50 U/μl), 0.15 μl of 100 mM dNTPs (with dTTP), 0.19 μl of RNase inhibitor (20 U/μl) and 4.16 μl of nuclease-free water. Subsequently, 500 ng of miRNA and 3μl of 1X looped-primers were added (specific for each microRNA), mixed well and incubated for 30 min at 16°C followed by 30 min at 42°C and 5 minutes at 85°C. The resultant cDNA was diluted to a minimum of 1:15 with nuclease free water and 1.33 μl was used in real-time PCR.

Real-time PCR was performed on Light cycler 480 II PCR System (PCR System, Roche Applied Science, Indianapolis, IN, USA). During the target amplification step, the AmpliTaq^®^ Gold DNA polymerase amplifies target cDNA synthesized from the RNA samples using sequence-specific primers from the TaqMan Assay. Real-time PCR reactions based on TaqMan^®^ reagent chemistry were performed in triplicate, and template controls were run for each assay under the same conditions. End-point PCR was performed in 20 μl reaction that contained 10 μl TaqMan 2X Universal PCR Master Mix No AmpErase UNG, 7.67 μl of nuclease free water, 1.33 μl diluted RT product (Minimum1:15) and 1 μl miRNA-specific PCR probe. The cycling conditions were as follows: 95°C for 10 minutes followed by 40 cycles consisting of 95°C for 15 seconds followed by 60°C for 60 seconds. The miRNA expression levels were normalized to the level of *RNU6B* for human samples and *U6* snRNA for mouse samples to correct for differential cDNA content.

### Synthesis of cDNA and real-time PCR

Total RNA was isolated, and the cDNA was synthesized by reverse transcription as previously described [26]. Briefly, reverse transcription of RNA was performed by adding 10 μl of (2 μg) total RNA, 1 μl of Oligo (dT)12–18 (500 μg/ml), and 1 μl of 10 mM dNTP (Invitrogen, Carlsbad, CA), incubating at 65°C for 5 minutes and immediately chilling on ice. Then, the master mix containing 4 μl of (5X) first strand RT buffer, 1 μl of 0.1 M DTT, and 1 μl of RNaseOUT (RNase Inhibitor) was incubated at 42°C for 2 min. Finally, 1 μl (50 units) of SuperScript II RT was added to each tube and incubated at 42°C for 50 minutes followed by 70°C for 15 minutes in order to destroy the superscript II RT.

The real-time primers for all the genes (human and mouse) were designed using Primer 3 software (Supplementary table S2). Real-time PCR was performed on Light cycler 480 II PCR System, (Roche Applied Science, Indianapolis, IN, USA). Real-time PCR reactions were performed in triplicate; non-template controls (NTCs) and a standard curve was run for each assay under similar conditions. Real-time PCR was performed in a 10 μl reaction containing 5 μl 2X SBYR green Master mix, 3.6 μl of nuclease free water, 1 μl diluted RT product (1:10), and 0.2 μl each of forward and reverse primer (5 pmol/μl). The cycling conditions used were as follows: 95°C for 10 minutes, followed by 40 cycles of 95°C for 15 seconds and 60°C for 1 minute. Gene expression levels were normalized to the level of β-actin expression and were reported relative to the expression level in RNA from corresponding normal controls.

### Generation of Let-7b overexpressing human PC cell lines

The 293FT cells were transfected with (2 μg) either with Let-7b over-expressing lentiviral pCDH-CMV-MCS-EF1-copGFP-Let-7b vector or with vector control and along with 10 μg of pPACKH1 Packaging Plasmid Mix (System Bioscience, CA, USA) using lipofectamine-2000 plus reagents (Invitrogen). The viral supernatant, collected after 48 and 72 hours post-transfection, was filtered and used to infect human PC cells (CD18/HPAF and Capan1) after mixing with 4 μg/ml of polybrene. The GFP+ cells were sorted by FACS, and Let-7b overexpression was analyzed by TaqMan assay.

### Immunoblot assay

Protein lysates were prepared from Let-7b-overexpressing and vector-transfected GFP positive cells, followed by Western blot analysis. Briefly, the cells from 70% confluency cultures were washed twice with PBS and lysed in RIPA buffer (50 mM Tris-HCl, pH 7.4; 0.25% Na-deoxycholate; 1 mM EDTA ;150 mM NaCl; 1% NP-40), supplemented with protease inhibitor cocktail, 5 mM Na3VO4, 5 mM NaF, and 1 mM phenylmethylsulphonyl fluoride. Protein lysates were quantified and resolved using 10% acrylamide/bisacrylamide gels (2% agarose gel for MUC4) and transferred onto a PVDF membrane. The membranes were blocked in 5% nonfat dry milk in PBS for at least 1 hour and then incubated with primary antibodies (anti-Kras, anti-MSST1, anti-MUC4, anti-caspase 9 anti-cyclin D1 and anti-β-actin) overnight at 4°C. Then, the membrane was washed (4X 10 min) with phosphate buffered saline and 0.1% Tween 20 (PBST) and probed with 1:5000 diluted horseradish peroxidase-conjugated anti-mouse or anti-rabbit secondary antibodies for 1 hour at room temperature. After washing the blots, the signal was detected with an ECL chemiluminescence kit (Amersham Bioscience, UK).

### Statistical analyses

The fold change in the miRNA expression was calculated by ΔΔCt method as a 2^−ΔΔCt^. U6 and RNU6B were used for normalization in mouse and human samples, respectively. A change of two-fold or more (on a log scale of ≥ 0.3) was considered statistically significant. The miRNA ratios of experimental samples to controls were compared to one using the one-sample non-parametric Wilcoxon sign rank test. The *p*-value was not adjusted for multiple comparisons. SAS software (SAS Institute Inc., Cary, NC) was used for data analysis.

## SUPPLEMENTARY TABLES




