# Supplementary material for: Changes in microRNA (miRNA) expression during pancreatic cancer development and progression in a genetically engineered KrasG12D;Pdx1-Cre mouse (KC) model
Source: Oncotarget. 2015 Oct 19;6(37):40295–309. doi: 10.18632/oncotarget.5641 (PMC4741896; doi:10.18632/oncotarget.5641)
Supplement: Supplementary file 1 [file oncotarget-06-40295-s001.pdf]

SUPPLEMENTARY TABLES

Supplementary Table S1: MicroRNA microarray data-global miRNA expression profiles at 25 weeks of mouse pancreatic cancer progression.

Supplementary Table S2: Real time primer sequences for human and mouse genes

S. No	Gene	Human	Mouse
1	DGCR8	F: 5′-GCTGACACCTTGGAAATGCT-3′	5′- CCTTCACATCCTGAGCAAGC -3′
		R: 5′-GACATCTTGGGCTTCTTTCG-3′	5′- ACTGTGCACAAGGGCTCAC -3′
2	TARBP2	F: 5′- CTCTGAGGAGCAGGCCTTT-3′	5′- TCTCTGAGGAGCAGGCTTTC -3′
		R: 5′- GCAGAGCCATGACACACAGT-3′	5′- CTGGTGGTTGCAGAACCATA -3′
3	Ago2	F: 5′- CCTGGTGGATAAGGAACATGA-3′	5′- AGAACATGACAGCGCTGAAG -3′
		R: 5′- CGCAGAGTGTCTTGGTGAAC-3′	5′- AAAGTACATGGTGCGCAGTG -3′
4	PRKRA	F: 5′- TTGCCAAGGAACAAGGTTTT-3′	5′- CTGAGTGCCAACGGACAGTA-3′
		R: 5′- ATACCGGAGCCATGACAGAC-3′	5′-ACTGCAGAGCATTGTGAGCA-3′
5	Dicer	F:5′-TGCGAGAATTGCTTGAAATG-3′	5′-ATTGCTCGAGATGGAACCAG-3′
		R: 5′-CTCTGACCTTCCCGTCGTAA-3′	5′-CACTTCCACGGTGACTCTGA-3′
6	Drosha	F: 5′-AATGGATGCGCTTGAAAAAT-3′	5′-TCAATGAACCGCTTCTGATG-3′
		R: 5′-TGATGCTCTCTTTCCCACCT-3′	5′-GGGAGAAAGGATAGGCTGTG-3′
7	Exportin-5	F: 5′-GACCAATTCAAACGCCTCAT-3′	5′-AATGGAGCAGATCCCTGAAA-3′
		R: 5′-CCAGCATTGGCTTTGTTTTT-3′	5′-TTCGGAACTGTTCTCCCAAG-3′
8	EGFR		5′-CTGCCGTGTCAAAGAAGACG-3′
			5′-CCTCTTGGGAACAGATTGGT-3′
9	TGFB1		5′-GAAGGACCTGGGTTGGAAGT-3′
			5′-TGGTTGTAGAGGGCAAGGAC-3′
10	Kras		5′-GAAAAGACTCCTGGCTGTGTG-3′
			5′-ATGTTTTCGAATTTCTCGGACT-3′
11	Bcl2		5′-CCTGGCTGTCTCTGAAGACC-3′
			5′-CATATTTGTTTGGGGCAGGT-3′
12	Galactin3		5′-TGGGGAAAGGAAGAAAGACA-3′
			5′-TGCAGTAGGTGAGCATCGTT-3′
13	CMYB		5′-TGTCCTCAAAGCCTTTACCG-3′
			5′-GTCCTCTGTCTTCCCACAGG-3′
14	E2F1		5′-ATCCCAGTCAATCCCTGTTG-3′
			5′-AACATGCTCCAGGAGTGAGTC-3′
15	Pten		5′-CGATCTTGACAAAGCAAACAA-3′
			5′-GTTGAACTGCTAGCCTCTGGA-3′

(*Continued*)

S. No	Gene	Human	Mouse
16	Fut8		5′-AAAATTCACTTCGGGGTGTG-3′
			5′-GATGCAGGGTTTGCATGATT-3′
17	Cdh11		5′-ACGCTGAAGCCTACATCCTG-3′
			5′-TCTTTTGCCTCCTCAGGGTA-3′
18	β-actin	F: 5′-AGTCCCTTGCCATCCTAAAAG-3′	5′-ACTGCTCTGGCTCCTAGCAC-3′
		R: 5′-CAATGCTATCACCTCCCCTG-3′	5′-ACATCTGCTGGAAGGTGGA-3′
