# Supplementary material for: Changes in microRNA (miRNA) expression during pancreatic cancer development and progression in a genetically engineered KrasG12D;Pdx1-Cre mouse (KC) model
Source: Oncotarget. 2015 Oct 19;6(37):40295–309. doi: 10.18632/oncotarget.5641 (PMC4741896; doi:10.18632/oncotarget.5641)
Supplement: Supplementary file 2 [file oncotarget-06-40295-s002.docx]

| **Supplementary table 1. MicroRNA microarray data-global miRNA expression profiles at 25 weeks of mouse pancreatic cancer progression** | | | | | | | |
| --- | --- | --- | --- | --- | --- | --- | --- |
| **Probe Set Name** | **Kras^G12D^;Pdx1-Cre (KC)** | **p-value (KC)** | **Detection** | **LSLKras^G12D^ control** | **p-value (LSLKras^G12D^)** | **Detection** | **Kras^G12D^;Pdx1-Cre/LSLKras^G12D^** |
| mmu-miR-150_st | 410.9821 | 2.05E-08 | TRUE | 2759.638 | 2.05E-08 | TRUE | 0.148926091 |
| mmu-miR-494_st | 599.1048 | 2.05E-08 | TRUE | 2040.199 | 2.05E-08 | TRUE | 0.293650178 |
| mmu-miR-138_st | 421.8911 | 2.05E-08 | TRUE | 1402.329 | 2.05E-08 | TRUE | 0.3008503 |
| mmu-miR-96_st | 24.11333 | 0.2196307 | FALSE | 70.00717 | 0.002954881 | TRUE | 0.344440862 |
| mmu-miR-148a-star_st | 62.99984 | 0.00109748 | TRUE | 179.2928 | 6.02E-06 | TRUE | 0.351379643 |
| mmu-miR-193_st | 94.40881 | 2.15E-05 | TRUE | 268.5935 | 1.91E-07 | TRUE | 0.351493279 |
| mmu-miR-451_st | 83.46148 | 9.86E-05 | TRUE | 228.405 | 7.15E-08 | TRUE | 0.365410039 |
| mmu-miR-216a_st | 2664.405 | 2.05E-08 | TRUE | 6566.258 | 2.05E-08 | TRUE | 0.405772207 |
| mmu-miR-29b_st | 209.366 | 3.83E-08 | TRUE | 511.2695 | 2.05E-08 | TRUE | 0.409502229 |
| mmu-miR-139-5p_st | 238.0682 | 2.05E-08 | TRUE | 553.8373 | 2.05E-08 | TRUE | 0.429852233 |
| mmu-miR-129-3p_st | 35.91602 | 0.01683308 | TRUE | 81.35419 | 0.001191286 | TRUE | 0.4414772 |
| mmu-miR-29c_st | 253.8189 | 5.52E-08 | TRUE | 567.4138 | 2.05E-08 | TRUE | 0.44732592 |
| mmu-miR-216b_st | 1635.343 | 2.05E-08 | TRUE | 3651.521 | 2.05E-08 | TRUE | 0.447852552 |
| mmu-miR-695_st | 33.64375 | 0.01276031 | TRUE | 74.49454 | 0.000835874 | TRUE | 0.451627059 |
| mmu-miR-483-star_st | 22.47902 | 0.1073228 | FALSE | 46.23727 | 0.03042611 | TRUE | 0.486166679 |
| mmu-miR-148a_st | 3081.672 | 2.05E-08 | TRUE | 6204.458 | 2.05E-08 | TRUE | 0.496686737 |
| mmu-miR-486_st | 89.08057 | 0.00019201 | TRUE | 176.7666 | 4.63E-05 | TRUE | 0.50394458 |
| mmu-miR-342-3p_st | 613.7523 | 2.05E-08 | TRUE | 1175.645 | 2.05E-08 | TRUE | 0.522055808 |
| mmu-miR-184_st | 85.37989 | 6.48E-05 | TRUE | 163.2611 | 7.36E-06 | TRUE | 0.522965299 |
| mmu-miR-188-5p_st | 24.14924 | 0.03971146 | TRUE | 45.42817 | 0.03101432 | TRUE | 0.531591741 |
| mmu-miR-1224_st | 3217.799 | 2.05E-08 | TRUE | 5988.312 | 2.05E-08 | TRUE | 0.537346584 |
| mmu-miR-150-star_st | 33.20552 | 0.0412836 | TRUE | 61.00882 | 0.003805016 | TRUE | 0.54427409 |
| mmu-miR-217_st | 3084.917 | 2.05E-08 | TRUE | 5622.202 | 2.05E-08 | TRUE | 0.548702626 |
| mmu-miR-15a_st | 660.2039 | 2.05E-08 | TRUE | 1200.576 | 2.05E-08 | TRUE | 0.549905962 |
| mmu-miR-375_st | 3288.907 | 2.05E-08 | TRUE | 5971.954 | 2.05E-08 | TRUE | 0.550725441 |
| mmu-miR-1195_st | 815.2899 | 2.05E-08 | TRUE | 1457.857 | 2.05E-08 | TRUE | 0.559238595 |
| mmu-miR-705_st | 270.3493 | 5.02E-07 | TRUE | 477.1687 | 1.47E-06 | TRUE | 0.566569643 |
| mmu-miR-141_st | 584.3497 | 2.05E-08 | TRUE | 1005.436 | 2.05E-08 | TRUE | 0.581190349 |
| mmu-miR-130b_st | 359.8303 | 2.47E-07 | TRUE | 617.7722 | 2.47E-07 | TRUE | 0.582464378 |
| mmu-miR-30e_st | 331.9242 | 3.83E-08 | TRUE | 568.8676 | 1.33E-07 | TRUE | 0.583482343 |
| mmu-miR-17-star_st | 183.2797 | 2.05E-08 | TRUE | 300.0377 | 3.83E-08 | TRUE | 0.610855569 |
| mmu-miR-802_st | 37.79035 | 0.01152761 | TRUE | 60.36853 | 0.000512968 | TRUE | 0.625994206 |
| mmu-miR-101b_st | 33.79184 | 0.0052051 | TRUE | 51.88671 | 0.002859328 | TRUE | 0.651261951 |
| mmu-miR-22-star_st | 32.71898 | 0.01768129 | TRUE | 49.08788 | 0.01669024 | TRUE | 0.666538869 |
| mmu-miR-155_st | 407.6113 | 2.05E-08 | TRUE | 607.4899 | 2.05E-08 | TRUE | 0.670976258 |
| mmu-miR-92a_st | 1004.058 | 2.05E-08 | TRUE | 1483.156 | 2.05E-08 | TRUE | 0.676973966 |
| mmu-miR-676_st | 810.8364 | 2.05E-08 | TRUE | 1159.062 | 2.05E-08 | TRUE | 0.699562577 |
| mmu-miR-691_st | 38.12562 | 0.04775452 | TRUE | 54.42955 | 0.006539828 | TRUE | 0.700458115 |
| mmu-miR-30a-star_st | 171.6486 | 5.52E-08 | TRUE | 244.1348 | 1.91E-07 | TRUE | 0.703089441 |
| mmu-miR-805_st | 382.4437 | 2.05E-08 | TRUE | 541.1696 | 1.33E-07 | TRUE | 0.706698418 |
| mmu-miR-455_st | 244.4583 | 2.05E-08 | TRUE | 344.901 | 3.83E-08 | TRUE | 0.708778171 |
| mmu-miR-193-star_st | 126.1322 | 4.32E-07 | TRUE | 174.7016 | 3.54E-07 | TRUE | 0.721986519 |
| mmu-miR-146a_st | 1054.529 | 2.05E-08 | TRUE | 1452.453 | 2.05E-08 | TRUE | 0.726033132 |
| mmu-miR-327_st | 37.81507 | 0.00737863 | TRUE | 51.67463 | 0.00483002 | TRUE | 0.73179179 |
| mmu-miR-466a-3p_st | 26.36959 | 0.05591316 | TRUE | 36.01437 | 0.02766183 | TRUE | 0.732196343 |
| mmu-miR-139-3p_st | 31.15559 | 0.05693374 | TRUE | 42.38807 | 0.01441326 | TRUE | 0.735008459 |
| mmu-miR-30b_st | 1558.044 | 2.05E-08 | TRUE | 2118.533 | 2.05E-08 | TRUE | 0.735435322 |
| mmu-miR-291b-5p_st | 29.29133 | 0.05668062 | TRUE | 39.55016 | 0.02079294 | TRUE | 0.740612175 |
| mmu-miR-182_st | 1348.785 | 2.05E-08 | TRUE | 1820.964 | 2.05E-08 | TRUE | 0.740698333 |
| mmu-miR-345-5p_st | 198.8394 | 2.86E-06 | TRUE | 268.3396 | 1.17E-05 | TRUE | 0.740999092 |
| mmu-miR-689_st | 54.35172 | 0.02967764 | TRUE | 73.31533 | 0.02525108 | TRUE | 0.741341818 |
| mmu-miR-32_st | 26.73125 | 0.05206034 | TRUE | 35.2794 | 0.009971802 | TRUE | 0.757701378 |
| mmu-miR-706_st | 133.6234 | 1.03E-06 | TRUE | 174.4273 | 2.28E-06 | TRUE | 0.766069302 |
| mmu-miR-672_st | 271.7648 | 2.05E-08 | TRUE | 336.8238 | 2.05E-08 | TRUE | 0.806845597 |
| mmu-miR-140-star_st | 980.4011 | 2.05E-08 | TRUE | 1214.728 | 2.05E-08 | TRUE | 0.807095169 |
| mmu-miR-187_st | 113.6031 | 0.00015218 | TRUE | 139.6391 | 5.63E-05 | TRUE | 0.813547925 |
| mmu-miR-149_st | 62.17022 | 0.00118325 | TRUE | 75.8634 | 0.001062908 | TRUE | 0.819502158 |
| mmu-miR-690_st | 4893.425 | 2.05E-08 | TRUE | 5885.76 | 2.05E-08 | TRUE | 0.831400703 |
| mmu-miR-20b_st | 446.1049 | 2.05E-08 | TRUE | 536.5195 | 2.05E-08 | TRUE | 0.831479378 |
| mmu-miR-30b-star_st | 24.43271 | 0.05738948 | TRUE | 29.29391 | 0.03522043 | TRUE | 0.834054245 |
| mmu-miR-378_st | 1507.541 | 2.05E-08 | TRUE | 1798.361 | 2.05E-08 | TRUE | 0.838286084 |
| mmu-miR-27b_st | 2481.569 | 2.05E-08 | TRUE | 2935.307 | 2.05E-08 | TRUE | 0.845420598 |
| mmu-miR-720_st | 681.1024 | 2.05E-08 | TRUE | 804.455 | 2.05E-08 | TRUE | 0.846663145 |
| mmu-miR-185_st | 682.2849 | 2.05E-08 | TRUE | 804.8135 | 2.05E-08 | TRUE | 0.847755287 |
| mmu-miR-1186_st | 31.0466 | 0.01885672 | TRUE | 36.35164 | 0.01384037 | TRUE | 0.854063255 |
| mmu-miR-290-5p_st | 27.41776 | 0.04435701 | TRUE | 32.06544 | 0.02452104 | TRUE | 0.85505641 |
| mmu-miR-30c_st | 2496.426 | 2.05E-08 | TRUE | 2888.722 | 2.05E-08 | TRUE | 0.864197386 |
| mmu-miR-29a_st | 4696.69 | 2.05E-08 | TRUE | 5395.451 | 2.05E-08 | TRUE | 0.870490715 |
| mmu-miR-425_st | 781.6047 | 2.05E-08 | TRUE | 895.7726 | 2.05E-08 | TRUE | 0.872548122 |
| mmu-miR-483_st | 64.36324 | 0.00225615 | TRUE | 73.54541 | 0.00039818 | TRUE | 0.875149652 |
| mmu-miR-152_st | 3917.324 | 2.05E-08 | TRUE | 4464.639 | 2.05E-08 | TRUE | 0.877411141 |
| mmu-miR-500_st | 351.7781 | 2.05E-08 | TRUE | 400.2354 | 2.05E-08 | TRUE | 0.878928001 |
| mmu-miR-423-3p_st | 166.9537 | 5.43E-06 | TRUE | 189.7376 | 2.51E-05 | TRUE | 0.879918899 |
| mmu-miR-16_st | 4985.843 | 2.05E-08 | TRUE | 5620.27 | 2.05E-08 | TRUE | 0.887118057 |
| mmu-miR-19b_st | 1975.553 | 2.05E-08 | TRUE | 2221.259 | 2.05E-08 | TRUE | 0.889384354 |
| mmu-miR-34a_st | 827.8589 | 2.05E-08 | TRUE | 928.8726 | 2.05E-08 | TRUE | 0.891251287 |
| mmu-miR-126-3p_st | 3127.758 | 2.05E-08 | TRUE | 3455.982 | 2.05E-08 | TRUE | 0.905027283 |
| mmu-miR-30a_st | 1879.758 | 2.05E-08 | TRUE | 2056.105 | 2.05E-08 | TRUE | 0.914232493 |
| mmu-miR-92b_st | 42.19337 | 0.00978676 | TRUE | 45.85673 | 0.008777229 | TRUE | 0.920112926 |
| mmu-miR-466e-3p_st | 26.33746 | 0.04861471 | TRUE | 28.57434 | 0.04375053 | TRUE | 0.921717177 |
| mmu-miR-148b_st | 89.75365 | 0.00015112 | TRUE | 97.29192 | 0.00020726 | TRUE | 0.922519054 |
| mmu-miR-15b_st | 1145.824 | 2.05E-08 | TRUE | 1235.4 | 2.05E-08 | TRUE | 0.92749231 |
| mmu-miR-203_st | 245.453 | 9.37E-07 | TRUE | 264.0748 | 2.47E-07 | TRUE | 0.929482859 |
| mmu-miR-17_st | 1670.593 | 2.05E-08 | TRUE | 1795.262 | 2.05E-08 | TRUE | 0.930556654 |
| mmu-miR-362-3p_st | 54.20742 | 0.00195071 | TRUE | 57.80706 | 0.00059247 | TRUE | 0.937730097 |
| mmu-miR-376b-star_st | 24.44442 | 0.03592218 | TRUE | 26.00613 | 0.01744132 | TRUE | 0.939948389 |
| mmu-miR-714_st | 53.01015 | 0.02432521 | TRUE | 56.12426 | 0.03783554 | TRUE | 0.944514012 |
| mmu-miR-362-5p_st | 231.681 | 7.27E-07 | TRUE | 243.5126 | 2.10E-06 | TRUE | 0.951412781 |
| mmu-let-7g_st | 1954.431 | 2.05E-08 | TRUE | 2033.634 | 2.05E-08 | TRUE | 0.961053464 |
| mmu-miR-23b_st | 4883.137 | 2.05E-08 | TRUE | 5061.521 | 2.05E-08 | TRUE | 0.964756839 |
| mmu-miR-709_st | 7970.234 | 2.05E-08 | TRUE | 8206.797 | 2.05E-08 | TRUE | 0.971174747 |
| mmu-miR-25_st | 700.4568 | 2.05E-08 | TRUE | 719.0386 | 2.05E-08 | TRUE | 0.974157437 |
| mmu-miR-106a_st | 592.9267 | 2.05E-08 | TRUE | 605.358 | 2.05E-08 | TRUE | 0.979464548 |
| mmu-miR-145_st | 4552.863 | 2.05E-08 | TRUE | 4636.957 | 2.05E-08 | TRUE | 0.981864399 |
| mmu-miR-324-3p_st | 36.2018 | 0.01147106 | TRUE | 36.78804 | 0.01517972 | TRUE | 0.984064386 |
| mmu-miR-181a-1-star_st | 27.23192 | 0.02784934 | TRUE | 27.67263 | 0.04608398 | TRUE | 0.984074156 |
| mmu-miR-1196_st | 144.8815 | 5.13E-06 | TRUE | 145.8567 | 3.99E-06 | TRUE | 0.993313986 |
| mmu-miR-7a_st | 50.03375 | 0.00101929 | TRUE | 50.30046 | 0.002013475 | TRUE | 0.994697663 |
| mmu-miR-106b_st | 974.5534 | 2.05E-08 | TRUE | 976.6268 | 2.05E-08 | TRUE | 0.997876978 |
| mmu-miR-26b_st | 107.0666 | 4.78E-06 | TRUE | 107.0291 | 8.11E-06 | TRUE | 1.000350372 |
| mmu-miR-143_st | 4650.255 | 2.05E-08 | TRUE | 4632.564 | 2.05E-08 | TRUE | 1.003818836 |
| mmu-miR-107_st | 2408.645 | 2.05E-08 | TRUE | 2336.478 | 2.05E-08 | TRUE | 1.030887087 |
| mmu-miR-106b-star_st | 115.0873 | 5.63E-05 | TRUE | 111.5547 | 0.000166236 | TRUE | 1.031666976 |
| mmu-let-7f_st | 1752.096 | 2.13E-08 | TRUE | 1696.704 | 2.13E-08 | TRUE | 1.032646826 |
| mmu-miR-762_st | 532.5922 | 5.03E-06 | TRUE | 512.1818 | 1.12E-05 | TRUE | 1.039849913 |
| mmu-miR-27a_st | 2761.941 | 2.05E-08 | TRUE | 2651.794 | 2.05E-08 | TRUE | 1.041536786 |
| mmu-let-7a_st | 7602.432 | 2.05E-08 | TRUE | 7289.037 | 2.05E-08 | TRUE | 1.042995392 |
| mmu-miR-200c_st | 5250.361 | 2.05E-08 | TRUE | 4981.949 | 2.05E-08 | TRUE | 1.053876906 |
| mmu-miR-26a_st | 7939.897 | 2.05E-08 | TRUE | 7477.531 | 2.05E-08 | TRUE | 1.061834047 |
| mmu-miR-103_st | 2744.187 | 2.05E-08 | TRUE | 2577.532 | 2.05E-08 | TRUE | 1.064656811 |
| mmu-miR-671-5p_st | 129.089 | 0.00032107 | TRUE | 120.9079 | 0.001727266 | TRUE | 1.0676639 |
| mmu-miR-93_st | 1366.811 | 2.05E-08 | TRUE | 1271.381 | 2.05E-08 | TRUE | 1.075060112 |
| mmu-miR-30d_st | 960.9976 | 2.05E-08 | TRUE | 885.5284 | 2.05E-08 | TRUE | 1.085225048 |
| mmu-miR-193b_st | 102.722 | 7.22E-05 | TRUE | 93.54588 | 0.000202964 | TRUE | 1.098092187 |
| mmu-miR-20a_st | 1751.252 | 2.05E-08 | TRUE | 1588.901 | 2.05E-08 | TRUE | 1.102178172 |
| mmu-miR-222_st | 685.6328 | 2.05E-08 | TRUE | 616.347 | 2.05E-08 | TRUE | 1.112413624 |
| mmu-miR-133a_st | 37.44796 | 0.00978066 | TRUE | 33.39703 | 0.03146971 | TRUE | 1.121296115 |
| mmu-miR-99a_st | 2684.43 | 2.05E-08 | TRUE | 2389.339 | 2.05E-08 | TRUE | 1.123503195 |
| mmu-miR-30c-2-star_st | 105.8911 | 1.86E-05 | TRUE | 93.93263 | 0.000173771 | TRUE | 1.127309009 |
| mmu-miR-877_st | 37.20009 | 0.00668995 | TRUE | 32.79887 | 0.02602444 | TRUE | 1.13418816 |
| mmu-miR-191_st | 3275.289 | 2.05E-08 | TRUE | 2883.729 | 2.05E-08 | TRUE | 1.135782523 |
| mmu-miR-181a_st | 1710.01 | 2.05E-08 | TRUE | 1504.072 | 2.05E-08 | TRUE | 1.136920307 |
| mmu-miR-340-5p_st | 44.10377 | 0.00752459 | TRUE | 38.27101 | 0.02456293 | TRUE | 1.152406743 |
| mmu-miR-652_st | 942.5152 | 2.05E-08 | TRUE | 814.5023 | 2.05E-08 | TRUE | 1.157167021 |
| mmu-miR-151-5p_st | 1229.749 | 2.05E-08 | TRUE | 1062.278 | 2.05E-08 | TRUE | 1.157652705 |
| mmu-let-7d_st | 7082.448 | 2.05E-08 | TRUE | 6098.754 | 2.05E-08 | TRUE | 1.161294258 |
| mmu-miR-493_st | 32.30517 | 0.02126965 | TRUE | 27.7772 | 0.05979949 | TRUE | 1.163010311 |
| mmu-miR-128_st | 52.25875 | 0.00102774 | TRUE | 44.86981 | 0.007272874 | TRUE | 1.16467509 |
| mmu-miR-574-3p_st | 425.024 | 2.05E-08 | TRUE | 362.8353 | 3.48E-06 | TRUE | 1.171396499 |
| mmu-miR-685_st | 342.4094 | 2.05E-08 | TRUE | 291.7013 | 2.05E-08 | TRUE | 1.173835701 |
| mmu-miR-140_st | 101.2105 | 3.87E-05 | TRUE | 85.60909 | 0.000167961 | TRUE | 1.18224011 |
| mmu-miR-700_st | 296.8764 | 1.33E-06 | TRUE | 250.7472 | 2.19E-05 | TRUE | 1.18396696 |
| mmu-miR-200a_st | 3758.279 | 2.05E-08 | TRUE | 3164.318 | 2.05E-08 | TRUE | 1.18770585 |
| mmu-miR-425-star_st | 52.42261 | 0.00204887 | TRUE | 44.10221 | 0.0195627 | TRUE | 1.188661747 |
| mmu-miR-23a_st | 5668.102 | 2.05E-08 | TRUE | 4746.964 | 2.05E-08 | TRUE | 1.194047817 |
| mmu-miR-200b-star_st | 461.0196 | 2.05E-08 | TRUE | 383.7068 | 2.05E-08 | TRUE | 1.201489262 |
| mmu-miR-141-star_st | 83.07054 | 0.00028582 | TRUE | 68.74752 | 0.00030388 | TRUE | 1.208342352 |
| mmu-let-7b_st | 9125.575 | 2.05E-08 | TRUE | 7438.814 | 2.05E-08 | TRUE | 1.226751334 |
| mmu-miR-125a-5p_st | 2318.336 | 2.05E-08 | TRUE | 1881.734 | 2.05E-08 | TRUE | 1.232021104 |
| mmu-miR-676-star_st | 55.23058 | 0.00072637 | TRUE | 44.70984 | 0.007562568 | TRUE | 1.235311511 |
| mmu-miR-532-3p_st | 77.15056 | 0.00032235 | TRUE | 61.8983 | 0.002154347 | TRUE | 1.246408383 |
| mmu-let-7i_st | 3938.124 | 2.05E-08 | TRUE | 3154.792 | 2.05E-08 | TRUE | 1.248299095 |
| mmu-miR-181b_st | 776.0866 | 2.05E-08 | TRUE | 618.0668 | 2.05E-08 | TRUE | 1.255667834 |
| mmu-miR-200b_st | 2777.181 | 2.05E-08 | TRUE | 2206.808 | 2.05E-08 | TRUE | 1.258460636 |
| mmu-miR-183-star_st | 40.6198 | 0.00156025 | TRUE | 32.14476 | 0.04023188 | TRUE | 1.263652303 |
| mmu-miR-711_st | 105.1086 | 0.00033883 | TRUE | 83.04578 | 0.000595362 | TRUE | 1.265670574 |
| mmu-miR-22_st | 4436.528 | 2.05E-08 | TRUE | 3502.805 | 2.05E-08 | TRUE | 1.266564368 |
| mmu-miR-24-2-star_st | 325.2733 | 2.05E-08 | TRUE | 250.1324 | 1.33E-07 | TRUE | 1.300404506 |
| mmu-miR-221_st | 1432.403 | 2.05E-08 | TRUE | 1091.242 | 2.05E-08 | TRUE | 1.312635511 |
| mmu-miR-24_st | 6411.076 | 2.05E-08 | TRUE | 4836.971 | 2.05E-08 | TRUE | 1.32543197 |
| mmu-miR-699_st | 59.04729 | 0.0026155 | TRUE | 44.15689 | 0.008621351 | TRUE | 1.337215778 |
| mmu-miR-378-star_st | 100.3442 | 0.00015164 | TRUE | 74.87891 | 0.001372603 | TRUE | 1.340086281 |
| mmu-miR-93-star_st | 47.2018 | 0.00346427 | TRUE | 34.89008 | 0.0141456 | TRUE | 1.352871647 |
| mmu-miR-28_st | 308.9988 | 2.47E-07 | TRUE | 227.8445 | 1.47E-06 | TRUE | 1.356182835 |
| mmu-miR-744_st | 202.4793 | 1.17E-05 | TRUE | 148.3752 | 8.90E-05 | TRUE | 1.364643822 |
| mmu-miR-29c-star_st | 36.14814 | 0.01545479 | TRUE | 26.36824 | 0.03756855 | TRUE | 1.370896958 |
| mmu-miR-574-5p_st | 65.66205 | 0.00047177 | TRUE | 47.78213 | 0.003492062 | TRUE | 1.374196797 |
| mmu-miR-100_st | 918.6657 | 2.05E-08 | TRUE | 666.3809 | 2.05E-08 | TRUE | 1.378589482 |
| mmu-let-7c_st | 12233.38 | 2.05E-08 | TRUE | 8820.966 | 2.05E-08 | TRUE | 1.386852642 |
| mmu-miR-346_st | 45.58416 | 0.00733786 | TRUE | 32.84397 | 0.05517315 | TRUE | 1.387900427 |
| mmu-miR-92a-star_st | 50.51538 | 0.00389993 | TRUE | 34.76495 | 0.0134566 | TRUE | 1.453054873 |
| mmu-miR-532-5p_st | 555.9703 | 2.05E-08 | TRUE | 382.5709 | 2.05E-08 | TRUE | 1.453247751 |
| mmu-let-7e_st | 3602.625 | 2.05E-08 | TRUE | 2457.713 | 2.05E-08 | TRUE | 1.465844466 |
| mmu-miR-99b_st | 1169.75 | 2.05E-08 | TRUE | 796.3177 | 1.34E-06 | TRUE | 1.468948888 |
| mmu-let-7b-star_st | 32.86008 | 0.02185156 | TRUE | 22.23154 | 0.08365797 | FALSE | 1.478083839 |
| mmu-miR-10b_st | 119.5405 | 2.08E-05 | TRUE | 80.06364 | 0.000516508 | TRUE | 1.493068514 |
| mmu-miR-130a_st | 874.6182 | 2.05E-08 | TRUE | 568.9879 | 7.15E-08 | TRUE | 1.537147275 |
| mmu-miR-429_st | 1570.526 | 2.05E-08 | TRUE | 992.9274 | 2.05E-08 | TRUE | 1.581712822 |
| mmu-miR-125b-5p_st | 6177.591 | 2.05E-08 | TRUE | 3873.221 | 2.05E-08 | TRUE | 1.594949268 |
| mmu-miR-721_st | 44.81172 | 0.00588365 | TRUE | 28.03937 | 0.04227459 | TRUE | 1.598171428 |
| mmu-miR-324-5p_st | 151.9021 | 1.96E-05 | TRUE | 94.2486 | 0.000383265 | TRUE | 1.611717309 |
| mmu-miR-299-star_st | 53.56485 | 0.00131447 | TRUE | 32.98567 | 0.02674196 | TRUE | 1.623882431 |
| mmu-miR-320_st | 658.0668 | 2.05E-08 | TRUE | 397.8347 | 2.05E-08 | TRUE | 1.654121171 |
| mmu-miR-339-5p_st | 105.3618 | 9.99E-05 | TRUE | 63.43296 | 0.007126744 | TRUE | 1.660994537 |
| mmu-miR-497_st | 828.4348 | 2.05E-08 | TRUE | 498.5688 | 2.05E-08 | TRUE | 1.661625838 |
| mmu-miR-18a_st | 248.6146 | 5.08E-07 | TRUE | 149.5988 | 5.36E-06 | TRUE | 1.66187563 |
| mmu-miR-181c_st | 158.2253 | 1.11E-05 | TRUE | 94.90507 | 0.000110427 | TRUE | 1.667195441 |
| mmu-miR-200a-star_st | 149.3293 | 2.05E-08 | TRUE | 89.27643 | 7.19E-05 | TRUE | 1.67266209 |
| mmu-miR-466f-3p_st | 53.94294 | 0.00234334 | TRUE | 31.66458 | 0.04845982 | TRUE | 1.70357352 |
| mmu-miR-19a_st | 76.88058 | 0.00010456 | TRUE | 45.1231 | 0.005281045 | TRUE | 1.703796503 |
| mmu-miR-210_st | 136.2804 | 3.30E-05 | TRUE | 79.82583 | 0.0020375 | TRUE | 1.707221835 |
| mmu-miR-183_st | 250.0105 | 1.03E-06 | TRUE | 145.3703 | 1.26E-05 | TRUE | 1.719818285 |
| mmu-miR-501-3p_st | 55.7295 | 0.00250337 | TRUE | 32.07103 | 0.02986307 | TRUE | 1.737689747 |
| mmu-miR-122_st | 102.9293 | 7.76E-05 | TRUE | 58.87611 | 0.002651603 | TRUE | 1.748235405 |
| mmu-miR-99b-star_st | 57.44814 | 0.00240489 | TRUE | 32.13558 | 0.05697342 | TRUE | 1.787680197 |
| mmu-miR-30e-star_st | 57.80788 | 0.00155961 | TRUE | 32.04911 | 0.05695916 | TRUE | 1.803728091 |
| mmu-miR-350_st | 58.35217 | 0.00200051 | TRUE | 32.25064 | 0.0234983 | TRUE | 1.809333706 |
| mmu-miR-151-3p_st | 180.0107 | 2.05E-08 | TRUE | 97.65568 | 1.65E-05 | TRUE | 1.843320327 |
| mmu-miR-423-5p_st | 70.99269 | 0.00078677 | TRUE | 37.05785 | 0.01946673 | TRUE | 1.915726088 |
| mmu-miR-1198_st | 82.68444 | 0.00046664 | TRUE | 42.7673 | 0.000975515 | TRUE | 1.93335656 |
| mmu-miR-674_st | 816.8937 | 2.05E-08 | TRUE | 421.856 | 2.05E-08 | TRUE | 1.936427833 |
| mmu-miR-195_st | 3205.333 | 2.05E-08 | TRUE | 1642.701 | 2.05E-08 | TRUE | 1.951257715 |
| mmu-miR-125a-3p_st | 63.31687 | 0.00069397 | TRUE | 31.37221 | 0.02626622 | TRUE | 2.018247041 |
| mmu-miR-28-star_st | 189.9806 | 2.05E-08 | TRUE | 92.77254 | 7.58E-05 | TRUE | 2.047810699 |
| mmu-miR-10a_st | 418.9965 | 1.33E-07 | TRUE | 199.3108 | 4.23E-06 | TRUE | 2.102226773 |
| mmu-miR-421_st | 60.65152 | 0.00062746 | TRUE | 28.1163 | 0.03711665 | TRUE | 2.157165772 |
| mmu-miR-335-5p_st | 111.3422 | 4.15E-06 | TRUE | 50.87277 | 0.000994029 | TRUE | 2.188640406 |
| mmu-miR-322-star_st | 65.97801 | 0.00033175 | TRUE | 29.91309 | 0.04887811 | TRUE | 2.205656788 |
| mmu-miR-345-3p_st | 69.44984 | 0.00053724 | TRUE | 31.25037 | 0.0145811 | TRUE | 2.222368567 |
| mmu-miR-708_st | 235.831 | 2.05E-08 | TRUE | 101.9162 | 0.000142657 | TRUE | 2.313969712 |
| mmu-miR-132_st | 285.0986 | 2.05E-08 | TRUE | 116.3978 | 3.91E-05 | TRUE | 2.449346981 |
| mmu-miR-329_st | 94.35875 | 6.76E-05 | TRUE | 35.97231 | 0.0218454 | TRUE | 2.623093985 |
| mmu-miR-337-5p_st | 114.9993 | 1.54E-06 | TRUE | 42.97707 | 0.004638168 | TRUE | 2.675829227 |
| mmu-miR-223_st | 113.282 | 5.02E-05 | TRUE | 41.46855 | 0.0149622 | TRUE | 2.731756958 |
| mmu-miR-300_st | 82.6607 | 5.25E-05 | TRUE | 28.73142 | 0.03183499 | TRUE | 2.877014084 |
| mmu-miR-127_st | 941.8656 | 2.05E-08 | TRUE | 325.5247 | 4.95E-06 | TRUE | 2.893376755 |
| mmu-miR-411_st | 158.2778 | 2.70E-06 | TRUE | 53.25666 | 0.009782288 | TRUE | 2.971981345 |
| mmu-miR-382_st | 127.9654 | 1.76E-06 | TRUE | 42.41294 | 0.004495419 | TRUE | 3.017131093 |
| mmu-miR-181d_st | 223.1902 | 1.04E-06 | TRUE | 70.81182 | 0.000784891 | TRUE | 3.151877751 |
| mmu-miR-199a-3p_st | 3744.417 | 2.05E-08 | TRUE | 1180.492 | 2.05E-08 | TRUE | 3.17191222 |
| mmu-miR-98_st | 74.82892 | 4.51E-05 | TRUE | 22.56417 | 0.0177368 | TRUE | 3.316271771 |
| mmu-miR-199b_st | 3672.523 | 2.05E-08 | TRUE | 1076.978 | 2.05E-08 | TRUE | 3.410026017 |
| mmu-miR-34c_st | 131.6047 | 2.10E-06 | TRUE | 38.45099 | 0.01574304 | TRUE | 3.422660899 |
| mmu-miR-199a-5p_st | 1813.095 | 2.05E-08 | TRUE | 523.146 | 2.05E-08 | TRUE | 3.465753346 |
| mmu-miR-199b-star_st | 227.1561 | 2.05E-08 | TRUE | 65.1668 | 0.000387868 | TRUE | 3.485764224 |
| mmu-miR-214_st | 850.6523 | 2.05E-08 | TRUE | 205.885 | 2.44E-05 | TRUE | 4.131686621 |
| mmu-miR-674-star_st | 131.2595 | 1.48E-07 | TRUE | 31.06214 | 0.01692743 | TRUE | 4.225706922 |
| mmu-miR-541_st | 264.2974 | 2.05E-08 | TRUE | 57.84825 | 0.000146538 | TRUE | 4.568805452 |
| mmu-miR-379_st | 590.6246 | 3.83E-08 | TRUE | 109.9913 | 9.83E-05 | TRUE | 5.369739243 |
| mmu-miR-21_st | 1062.33 | 2.05E-08 | TRUE | 184.5411 | 9.37E-07 | TRUE | 5.756603813 |
| mmu-miR-194_st | 5744.131 | 2.05E-08 | TRUE | 808.3148 | 1.33E-07 | TRUE | 7.106304375 |
| mmu-miR-192_st | 3412.096 | 2.05E-08 | TRUE | 408.5368 | 3.54E-07 | TRUE | 8.351991791 |
| mmu-miR-31_st | 2244.656 | 2.05E-08 | TRUE | 236.2369 | 1.03E-07 | TRUE | 9.501716286 |
| mmu-miR-205_st | 318.9843 | 2.05E-08 | TRUE | 32.24027 | 0.05296016 | TRUE | 9.893971111 |
| mmu-miR-146b_st | 713.3904 | 2.05E-08 | TRUE | 38.63734 | 0.004921975 | TRUE | 18.46375553 |
